# GIS-Enhanced Survey of Potential *Aedes aegypti* and *Aedes albopictus* Artificial Oviposition Containers Distributed across Communities in Trinidad, West Indies

**DOI:** 10.3390/insects15100779

**Published:** 2024-10-08

**Authors:** Limb K. Hapairai, Roshan Seeramsingh, Lester D. James, Rachel S. Feng, Naresh Nandram, Azad Mohammed, Molly Duman-Scheel, David W. Severson

**Affiliations:** 1 Department of Medical and Molecular Genetics, Indiana University School of Medicine, South Bend, IN 46617, USA; limbh@pihoa.org (L.K.H.); mscheel@nd.edu (M.D.-S.); 2 Eck Institute for Global Health, University of Notre Dame, Notre Dame, IN 46556, USA; 3 Insect Vector Control Division, Ministry of Health, Port-of-Spain 101002, Trinidad and Tobago; roshan.seeramsingh@health.gov.tt (R.S.); naresh.nandram@gmail.com (N.N.); 4 Department of Life Sciences, University of the West Indies, St. Augustine 685509, Trinidad and Tobago; lesterdjames@gmail.com (L.D.J.); shuifengr@gmail.com (R.S.F.); azad.mohammed@sta.uwi.edu (A.M.); 5 Department of Biological Sciences, University of Notre Dame, Notre Dame, IN 46556, USA

**Keywords:** mosquito control, arbovirus, dengue, GIS mapping, water storage

## Abstract

**Simple Summary:**

Household surveys of communities for potential oviposition containers by mosquito control teams offer effective mechanisms for reducing arbovirus transmission. Here, we combined standard community surveys with GIS mapping to identify premises with *Aedes*-positive containers and classify container types near the end of the dry season in Trinidad, West Indies. The entomological indices based on key containers (tanks, drums, tubs/basins/buckets) showed that two of the four locations surveyed were already at a high risk for arbovirus transmission going into the wet season. GIS mapping provided inspection teams with specific locations that had *Aedes*-positive containers and highlighted premises that were not accessible. This information can facilitate the identification of areas within communities that may be at greater risk and should be prioritized for future surveys and control efforts; it also highlights the need to inform and increase community participation.

**Abstract:**

Dengue and other arboviruses remain a global threat, and enhanced efforts to control the mosquitoes that transmit them are urgently needed. A survey of potential manmade *Aedes aegypti* (L.) and *Aedes albopictus* (Skuse) oviposition containers was performed in four communities near the end of the typical dry season in 2018 in Trinidad, West Indies. The purpose was to conduct individual premise surveys and use GIS mapping to visualize premises within communities that had *Aedes*-positive containers, as this information could be used for the prioritization of mosquito control efforts in potential high risk areas as the wet season progressed. Accessible premises were surveyed following standard inspection protocols used by the Insect Vector Control Division (IVCD), Ministry of Health (MOH). The results indicated that two of the four locations would be at high risk for arbovirus transmission going into the wet season. The GIS mapping of premises with *Aedes*-positive containers facilitated the identification of potential hot spots for arbovirus transmission risk within communities that should be prioritized for enhanced monitoring and vector control efforts, emphasizing the need to increase community participation in standard surveys by IVCD.

## 1. Introduction

The annual number of reported cases of dengue infection and the cases of emerging mosquito borne diseases transmitted by *Aedes aegypti* (L.) and *Aedes albopictus* (Skuse) (Diptera: Culicidae) continue to increase across the globe [1]. The availability of effective licensed vaccines for dengue, Zika, or chikungunya (CHIK) viruses is limited [2,3,4]. As such, controlling the spread of these diseases mainly relies on mosquito reduction through vector control strategies. Routine mosquito control efforts by the Insect Vector Control Division, Ministry of Health (IVCD/MOH) across Trinidad, West Indies, with an emphasis on source reduction and larvicidal treatment of potential *Ae. aegypti* oviposition sites [5,6], have been conducted for decades in efforts to reduce dengue transmission. In addition, *Ae. albopictus* was first reported in northwest Trinidad in 2003 [7] and has continued to expand its range across the island [8,9]. Still, while oviposition has been reported throughout the entire year [10], the transmission of dengue continues to occur primarily during the wet season, and a CHIK outbreak was reported in 2014, followed by a Zika outbreak in 2016 [11,12].

Unregulated urban growth, which is often accompanied by the lack of or unreliability of municipal water supplies, promotes water storage and subsequently encourages oviposition in water storage containers and the proliferation of *Aedes* mosquitoes [13]. Previous *Aedes* larval and pupal surveys in Trinidad indicated that outdoor tanks, drums, and tubs/basins/buckets near homes were the primary sources of *Aedes* adult production [5,14]. Historically, residents have often depended on metal or plastic drums for the storage of municipal or rain water for laundry, drinking, bathing, and household needs [15]. Although access to municipal water has expanded, many urban residents now utilize large plastic tanks (typically >3700 l) to store water in the event the municipal water supply fails [8]. These are easier to cover properly to prevent mosquito oviposition, but can be highly productive breeding sites if not properly secured [14].

Geographical Information System (GIS) technology offers innovative tools to visualize spatial patterns of entomological data to bolster public health capacity in managing vector-borne diseases in resource limited environments [16,17]. Successful larval control is often the result of the combination of removal and the effective larvicidal treatment of identified potential oviposition containers. The results of simple surveys across communities can easily be recorded using handheld GPS receivers. This could facilitate the rapid transfer of information to an electronic data base for GIS mapping and visualization [18] to improve the efficacy and accuracy of vector control operations [19]. Although commonly used entomological indices for monitoring *Aedes* populations often show limited correlation with predicting risk of arbovirus transmission at the household level [20], this information could be used to identify and prioritize potential household clusters at a high risk for arbovirus transmission across communities at the beginning of and throughout the wet season. The purpose of these surveys was to conduct standard IVCD surveys to identify the presence and frequency of potential *Aedes* oviposition containers within individual premises in communities in Trinidad, and then use GIS to visualize the distribution of premises with *Aedes*-positive containers. This would allow IVCD staff to prioritize these areas, including the expansion of efforts to increase community awareness and participation in *Aedes* control efforts. 

## 2. Materials and Methods

### 2.1. Study Sites

**Figure 1:**
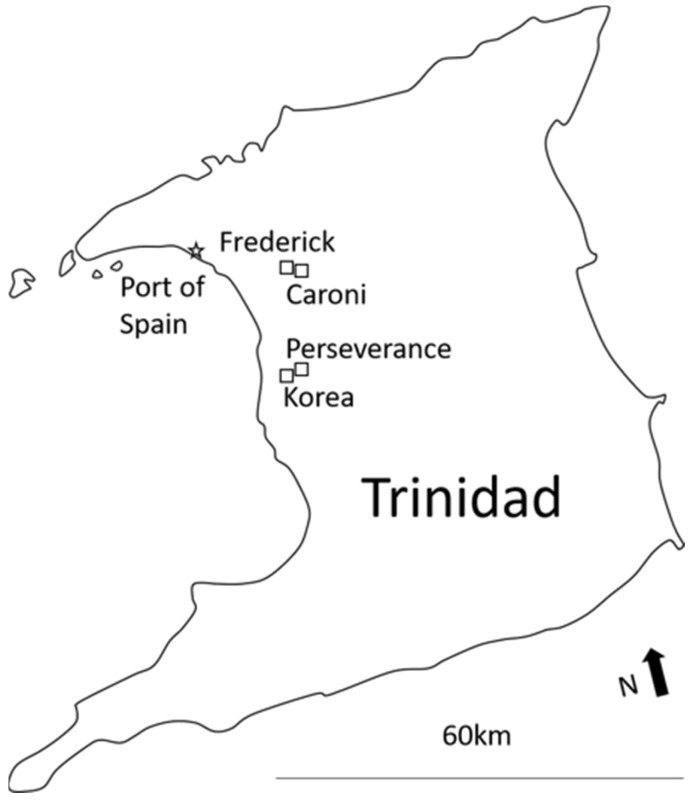
Locations of survey sites for potential *Aedes* breeding containers in Trinidad, West Indies.

Locations were selected based on satellite images that allowed for identification of residential areas surrounded by either natural (open savanna and/or forest areas) or man-made barriers (large commercial/industrial areas) within the county of Caroni in northwest Trinidad. Our rationale was that such areas appear to have similar characteristics in terms of socio-economic levels and government services like municipal water and waste collection, and would likely have minimal *Aedes* migration and immigration that might confound future efforts by IVCD and collaborators to investigate the efficacy of current and novel control efforts. Entomological surveys were thereafter conducted once per household during 2018 in four communities that met these criteria: Frederick Settlement (25–26 April; 10°36′17″ N 61°23′40″ W), Caroni Settlement (9–10 May; 10°36′21″ N 61°22′56″ W), Perseverance Village (15–16 May; 10°29′36″ N 61°25′38″ W), and Korea Village (23–24 May, 10°29′07″ N 61°25′55″ W) (Figure 1). This time period represents the end of the typical dry season (December–May) in Trinidad [21] and was chosen to provide information on locations that might be at higher risk for *Aedes* breeding and arbovirus transmission during the upcoming wet season (May–November). These communities, including particular areas within them, would represent priorities for vector control efforts by IVCD during the upcoming rainy season and could also be good targets for testing novel larvicides.

### 2.2. Survey Methods

Surveys were conducted to assess the abundance and locations of potential manmade oviposition sites following the standard house-to-house inspection protocol [5,22] utilized by IVCD staff. Inspection teams were trained on the use of handheld GPS units that were then used for these surveys. For this, all manmade containers that could hold water and were located around the exterior of each premise were identified, counted, and recorded. These were subsequently characterized into six basic categories including: tanks, drums, tubs/basins/buckets, tires, plant saucers/vases, and small miscellaneous containers [14]. Those containing water were further inspected to determine presence or absence of mosquito larvae or pupae. Up to 20 larvae and/or pupae per positive container were collected, subsequently reared to adulthood using a standard protocol [23], and identified to mosquito species at the UWI insectary using Darsie and Ward [24]. Any containers with remaining larvae or pupae were emptied or treated with Aquatain^®^ Mosquito Formulation by IVCD staff [25]. 

Satellite images were obtained from Google Earth, and images indicating the distributions of the potential oviposition sites were generated using ESRI ArcMap software version 10.2. All premises that were surveyed were mapped, and those with at least one container positive for *Aedes* juveniles were indicated. In addition, all premises surveyed were identified as follows: (1) those with at least one key container category; (2) those that did not have any of the three key container categories (larger volume containers like tanks, drums, and tubs/basins/buckets); and (3) those that did not have any of the three other container categories (smaller volume containers like tires, plant saucers/vases, and small miscellaneous) [14]. 

### 2.3. Entomological Indices

Two entomological indices used to evaluate and compare data for *Aedes*-positive premises across the four communities were calculated from survey data: the House index (HI: percentage of houses with at least one container positive for larvae and/or pupae) and the Breteau index (BI: number of containers positive for larvae and/or pupae per 100 houses inspected) [26]. However, these indices, as epidemiological indicators of the potential for dengue transmission, fail to account for the potential numbers of adult mosquitoes produced by individual container types. Therefore, we also calculated these indices based solely on the number of key containers with at least one positive for larvae and/or pupae (tanks, drums, tubs/basins/buckets) that are known to be the most productive container types, being reported for producing as high as 90% of the total larvae/pupae observed during container surveys of individual households, which are referred to here as HIkey and BIkey, respectively [5,14,22]. The rationale for calculating these indices was that this information might have the best potential to identify individual communities at greatest risk for large *Aedes* populations and associated arbovirus transmission risk prior to and thereafter during the typical wet season. 

## 3. Results

**Figure 2:**
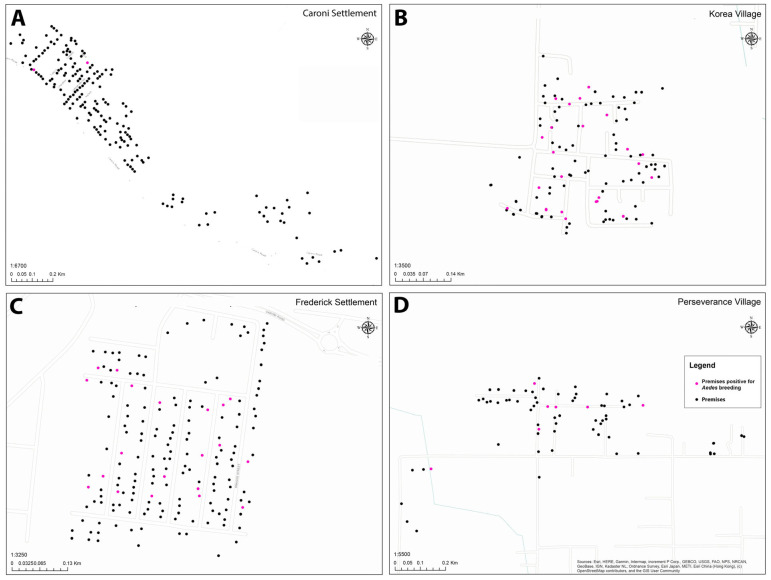
GIS mapping of the spatial distribution of premises surveyed. (**A**) Caroni Settlement. (**B**) Frederick Settlement. (**C**) Korea Village. (**D**) Perseverance Village. Premises with containers positive for larvae/pupae are highlighted (pink).

A total of 1011 residential and commercial premises were identified across all four locations. However, only 545 of these premises were accessible for inspection (55.9%) by IVCD or UWI crews over the survey period, largely because of owner absence during regular weekday working hours (Table 1). Access ranged from 48.6% (Caroni) to 69.1% (Korea Village). All premises inspected had at least one potential *Aedes* manmade oviposition container, and all adults obtained from larvae and pupae were identified, through subsequent rearing to adulthood at the insectary, to be *Ae. aegypti.* The GIS mapping of the premises surveyed facilitated the visualization of these across the entire community and highlighted the distribution of premises that had containers with active *Aedes* breeding (Figure 2). 

A total of 10,960 potential *Aedes* oviposition containers were identified, of which 120 (1.0%) were positive for *Ae. aegypti* larvae and pupae (Table 2). The largest number of containers was found in Caroni Settlement (3596) but only two were (0.05%) positive. The smallest number of containers were found in Perseverance Village (1527), but 15 of these were (0.98%) positive. Multiple containers were present in every residence with a maximum per individual premise of one hundred twenty-one (Korea Village) and a minimum of two (Frederick Settlement). Among the positive containers, the most frequent positive containers were drums (4.96%), plant saucers/vases (3.37%), and tires (2.86%), with oviposition in tubs/basins (0.94%), tanks (0.64%), and small misc. (0.63%) being less frequent. Further, only a small portion of premises across the four locations did not have at least one positive container of the key container types (1.4–2.7%) or at least one positive container of the other container types (1.8–5.1%) (Table 3). 

Entomological indices used to assess the presence of containers and those with larvae/pupae were calculated, with Korea Village having the highest HI (21.4) and BI (42.9) indices, while Caroni Settlement had the lowest HI (1.1) and BI (1.1) indices (Table 3). Relative to key containers only, Korea Village also had the highest HIkey (13.4) and BIkey (23.3) indices observed across all four sites.

## 4. Discussion

In this study, individual household premises in four isolated communities in Trinidad, West Indies were physically surveyed to identify the presence of potential manmade *Aedes* oviposition containers in concert with GIS mapping to visualize the distribution of premises surveyed within individual communities. These premises surveys indicated that every household had multiple containers present that could serve as *Aedes* oviposition sites during the rainy season. Although the surveys were conducted during the late stages of the typical dry season, we did confirm active oviposition in 1.0% of the total containers identified. The inclusion of GIS mapping allowed for easy identification of premises with active mosquito breeding. 

The diversity of containers observed remained fairly consistent with previous surveys in Trinidad going back decades [5,15,21,22]. The entomological indices were highly variable across the four communities ranging from HI:BI:HIkey:BIkey of 1.1:1.1:0.5:0.5 (Caroni Settlement) to 21.4:42.9:13.4:23.4 (Korea Village) (Table 3). The indices for Korea Village would be considered very high for any time of year, but especially so for what is considered to be the dry season when mosquito breeding would be expected to be low. Further, when based on key containers, both Frederick Settlement (HIkey > 5.0) and Korea Village (HIkey > 5.0 and BIkey > 20) would be considered high priority (Priority II) risks for arbovirus transmission [26]. Although individual entomological indices have been shown to have limited utility for predicting individual household risks for arbovirus infection, they have been shown (particularly the BI) to be predictive for risk at the household cluster level [20]. This is likely due to *Aedes* production and arbovirus transmission at non-residential areas, wherein individuals are exposed to the virus while conducting regular daily activities outside the residential area at sites like work, schools, and other public sites [27,28]. Once introduced, arbovirus transmission can rapidly expand within the immediate neighborhood and beyond due to house-to-house movement of infected people during regular daily activities [29]. Of note, both Frederick Settlement and Korea Village had recently or currently experienced a higher level of unregulated peripheral settlement (squatting) as a result of rapid urban growth. This suggests that late dry season community surveys and GIS mapping could identify those with the potential for very high transmission during the pending wet season and these could be prioritized for enhanced monitoring and the implementation of vector control efforts by IVCD. 

Of note we only identified *Ae. aegypti* from larvae/pupae collected in containers during our surveys. We anticipated finding *Ae. albopictus*, as it is known to be present across Trinidad [8,9] since first being recorded in December 2002 at Chaguaramus in the far northwest region [7]. Driven largely by human activities, *Ae. albopictus* has expanded its range around the world, resulting in an increased risk of dengue transmission [26,27]. *Ae. aegypti* and *Ae. albopictus* often coexist, with *Ae. albopictus* generally being more prevalent in peri-urban and sylvatic environments [30,31,32,33,34]. Previous seasonal observations suggest that *Ae. aegypti* is more predominant in Trinidad [8,9] and Florida [35] until later in the rainy season, when *Ae. albopictus* densities often increase. These studies showed that seasonal distributions of the two species generally reflect life history differences between them, wherein *Ae. aegypti* survives better in drier and hotter microclimates, while *Ae. albopictus* is more competitive in wetter and more humid microclimates.

Our results also highlight the difficulty in gaining widescale access to individual premises for *Aedes* inspection and the subsequent application of breeding source reduction and treatment technologies during a typical work day. Our inability to access premises across large proportions of individual communities would limit attempts at the widespread deployment of novel larvicides or adulticides to reduce *Aedes* populations to prevent associated arbovirus transmission. This appears to be a long-standing problem in Trinidad, as the routine focal insecticide treatment of water holding containers by IVCD staff is known to be inconsistent across communities due to the lack of access to a high proportion of premises [36]. While we encountered a minimal lack of access due to homeowner rejection of our request, the major problem was due to homeowner absence, likely due to employment during normal work days. Although efforts to access premises would potentially be enhanced by performing surveillance/control efforts after typical working hours, the associated time constraints could compromise the ability to inspect/treat whole communities. 

The inability to access premises across individual communities highlights the need to actively encourage community engagement in coordination with research planning for the deployment of lethal ovitraps or other larvicidal/adulticidal mosquito control efforts. We have since conducted multiple community engagement activities across communities in Trinidad via open community forums and paper surveys [37,38]. Most participants were highly supportive of larviciding and lethal ovitrap deployment around their premises as biorational *Aedes* control tools. They also offered suggestions for enhancing operational approaches to facilitate community-wide deployment. The need for educational campaigns to inform community stakeholders about mosquito vector biology and management was considered a priority. Of note, a previous study that deployed autocidal gravid ovitraps (AGO) across two communities in Puerto Rico was highly successful in significantly reducing adult *Aedes* female populations around target houses [39]. They reported successful deployment of AGO traps within 78% and 84% of the premises in the two communities. This likely represents a minimum threshold for lethal ovitrap deployment efforts in future studies. 

The successful combined utilization of both standard premise inspection and GIS-mediated oviposition container surveillance in Trinidad provides further evidence that including GIS technology can benefit resource-limited vector control divisions. Staff training was critical, but once trained, the staff members successfully utilized the GPS units, and all staff members collected GPS data while completing routine surveillance operations. The results of this study indicate that investments in GIS technology can be worthwhile and could help support mosquito control during the rainy season, when vector control divisions can more easily direct their efforts to hotspots identified during the dry season and perhaps thereby minimize mosquito population growth and expansion. 

## Figures and Tables

**Table 1:** Survey results for presence of *Aedes* larvae and/or pupae in premises across four locations in Trinidad, West Indies, conducted between 25 April and 24 May 2018.

Location	Total No. Premises	No. Premises Inspected	Percent Premises Inspected
Caroni Settlement	385	187	48.6%
Frederick Settlement	329	175	53.2%
Perseverance Village	135	71	52.6%
Korea Village	162	112	69.1%
Total	1011	545	53.9%

**Table 2:** Survey results for number (#) of potential oviposition container types and identification of containers positive (+) for presence of *Aedes aegypti* larvae and/or pupae on premises across four locations in Trinidad, West Indies, conducted between 25 April and 24 May 2018.

Container Type	Caroni Settlement		Frederick Settlement		Perseverance Village		Korea Village		Total			
	#	+	#	+	#	+	#	+	#	#%	+	+%
Tanks	359	0	313	4	136	1	135	1	943	8.60	6	0.64
Drums	174	1	148	9	50	3	132	12	504	4.60	25	4.96
Tubs/Basins/Buckets	928	0	869	14	425	0	647	13	2869	26.18	27	0.94
Tires	163	1	73	2	63	2	156	8	455	4.15	13	2.86
Plant Saucers/Vases	89	0	82	12	95	0	90	0	356	3.25	12	3.37
Small Misc.	1883	0	1782	14	758	9	1410	14	5833	53.17	37	0.63
Total	3596	2	3267	55	1527	15	2570	48	10,960		120	

**Table 3:** Entomological indices for *Aedes aegypti* across four locations in Trinidad, West Indies, conducted between 25 April and 24 May 2018.

Location	HI ^a^	BI ^b^	HIkey ^c^	BIkey ^d^	Premises with No Key Containers (%)	Premises with No Misc. Containers (%)
Caroni Settlement	1.1	1.1	0.5	0.5	2.7	3.7
Frederick Settlement	14.3	31.4	8.0	10.9	2.3	5.1
Perseverance Village	9.9	21.1	4.2	5.6	1.4	2.8
Korea Village	21.4	42.9	13.4	23.3	2.7	2.7

^a^ House index (HI). ^b^ Breteau index (BI). ^c^ House index for key containers only (HIkey). ^d^ Breteau index for key containers only (BIkey).

## Data Availability

The raw data supporting the conclusions of this article will be made available by the authors upon request and are subject to approval by the Insect Vector Control Division, Ministry of Health, Cunupia, Trinidad, West Indies.

## References

[1] WHO Dengue—Global Situation. https://www.who.int/emergencies/disease-outbreak-news/item/2024-DON518.

[2] Silva J.P., Fernandez-Sesma A. (2023). Challenges on the development of a dengue vaccine: A comprehensive review of the state of the art. J. Gen. Virol..

[3] Medina-Magues L.G., Gergen J., Jasny E., Petsch B., Lopera-Madrid J., Medina-Magues E.S., Salas-Quinchucua C., Osorio J.E. (2021). mRNA vaccine protects against Zika virus. Vaccines.

[4] Rezza G., Weaver S.C. (2019). Chikungunya as a paradigm for emerging viral diseases: Evaluating disease impact and hurdles to vaccine development. PLoS Negl. Trop. Dis..

[5] Chadee D.D. (2004). Key premises, a guide to *Aedes aegypti* (Diptera: Culicidae) surveillance and control. Bull. Entomol. Res..

[6] Chadee D.D. (2009). Oviposition strategies adopted by gravid *Aedes aegypti* (L.) (Diptera: Culicidae) as detected by ovitraps in Trinidad, West Indies (2002–2006). Acta Trop..

[7] Chadee D.D., Fat F.H., Persad R.C. (2003). First record of *Aedes albopictus* from Trinidad, West Indies. J. Am. Mosq. Control Assoc..

[8] James L.D., Winter N., Stewart A.T.M., Feng R.S., Nandram N., Mohammed A., Duman-Scheel M., Romero-Severson E., Severson D.W. (2022). Field trials reveal the complexities of deploying and evaluating the impacts of yeast-baited ovitraps on *Aedes* mosquito densities in Trinidad, West Indies. Sci. Rep..

[9] Hapairai L.K., Mysore K., James L.D., Scheel N.D., Realey J.S., Sun L., Gerber L.E., Feng R.S., Romero-Severson E., Mohammed A. (2021). Evaluation of large volume yeast interfering RNA lure-and-kill ovitraps for attraction and control of *Aedes* mosquitoes. Med. Vet. Entomol..

[10] Chadee D.D., Corbet P.S. (1987). Seasonal incidence and diel patterns of oviposition in the field of the mosquito, *Aedes aegypti* (L.) (Diptera: Culicidae) in Trinidad, West Indies: A preliminary study. Ann. Trop. Med. Parasit..

[11] Gonzalez-Escobar G., Churaman C., Rampersadd C., Singh R., Nathaniel S. (2021). Mayaro virus detection in patients from rural and urban areas of Trinidad and Tobago during the chikungunya and Zika virus outbreaks. Pathog. Glob. Health.

[12] Christie C.D.C., Lue A.M., Melbourne-Chambers R.H. (2023). Dengue, chikungunya and zika arbovirus infections in Caribbean children. Curr. Opin. Pediatr..

[13] Barrera R., Avila J., Gonzalez-Tellez S. (1993). Unreliable supply of potable water and elevated *Aedes aegypti* larval indices: A causal relationship?. J. Am. Mosq. Control Assoc..

[14] Chadee D.D., Doon R., Severson D.W. (2007). Surveillance of dengue fever cases using a novel *Aedes aegypti* population sampling method in Trinidad, West Indies: The cardinal points approach. Acta Trop..

[15] Chadee D.D., Rahaman A. (2000). Use of water drums by humans and *Aedes aegypti* in Trinidad. J. Vector Ecol..

[16] Lozano-Fuentes S., Elizondo-Quiroga D., Farfan-Ale J.A., Loroño-Pino M.A., Garcia-Rejon J., Gomez-Carro S., Lira-Zumbardo V., Najera-Vazquez R., Fernandez-Salas I., Calderson-Martinez J. (2008). Use of Google EarthTM to strengthen public health capacity and facilitate management of vector-borne diseases in resource-poor environments. Bull. World Health Org..

[17] Eisen L., Lozano-Fuentes S.S. (2009). Use of mapping and spatial and space-time modeling approaches in operational control of *Aedes aegypti* and dengue. PLoS Negl. Trop. Dis..

[18] Ai-Leen G.T., Jin Song R. (2000). The use of GIS in ovitrap monitoring for dengue control in Singapore. Dengue Bull..

[19] Chansang C., Kittayapong P. (2007). Application of mosquito sampling count and geospatial methods to improve dengue vector surveillance. Am. J. Trop. Med. Hyg..

[20] Morales-Perez A., Nava-Aguilera E., Hernandez-Alvarez C., Alvarado-Castro V.M., Arostegui J., Legorreta-Soberanis J., Flores-Moreno M., Morales-Nava L., Harris E., Ledogar R.J. (2020). Utility of entomological indices for predicting transmission of dengue virus: Secondary analysis of data from the Camino Verde trial in Mexico and Nicaragua. PLoS Negl. Trop. Dis..

[21] Chadee D.D. (1992). Seasonal incidence and horizontal distribution patterns of oviposition by *Aedes aegypti* in an urban environment in Trinidad, West Indies. J. Am. Mosq. Control Assoc..

[22] Focks D.A., Chadee D.D. (1997). Pupal survey: An epidemiologically significant surveillance method for *Aedes aegypti*: An example using data from Trinidad. Am. J. Trop. Med. Hyg..

[23] Clemons A., Mori A., Haugen M., Severson D., Duman-Scheel M. (2010). *Aedes aegypti*: Culturing and egg collection. Cold Spring Harb. Protoc..

[24] Darsie R.F., Ward R.A. (1981). Identification and geographical distribution of the mosquitoes of North America, north of Mexico. Mosq. Syst. Supp..

[25] Webb C.E., Russell R.C. (2009). A laboratory investigation of the mosquito control potential of the monomolecular film Aquatain^®^ mosquito formula against immature stages of *Aedes aegypti* and *Culex quinquefasciatus*. J. Am. Mosq. Control Assoc..

[26] WHO (2003). Guidelines for Dengue Surveillance and Mosquito Control.

[27] Morrison A.C., Getis A., Santiago M., Rigau-Perez J.G., Rieter P. (1998). Exploratory space-time analysis of reported dengue cases during an outbreak in Florida, Puerto Rico, 1991–1992. Am. J. Trop. Med. Hyg..

[28] Reiner R.C., Stoddard S.T., Scott T.W. (2014). Socially structured human movement shapes dengue transmission despite the diffusive effect of mosquito dispersal. Epidemics.

[29] Stodddard S.T., Forshey B.M., Morrison A.C., Paz-Soldan V.A., Vazquez-Prolopec G.M., Astete H., Reiner R.C., Vilcarromero S., Elder J.P., Halsey E.S. (2013). House-to-house human movement drives dengue virus transmission. Proc. Natl. Acad. Sci. USA.

[30] Kraemer M.U.G., Reiner R.C., Brady O.J., Messina J.P., Gilbert M., Pigott D.M. (2019). Past and future spread of the arbovirus vectors *Aedes aegypti* and *Aedes albopictus*. Nat. Microbiol..

[31] Messina J.P., Brady O.J., Golding N., Moritz U.G., Kraemer M.U.G., Wint G.R.W., Ray S.E., Pigott D.M., Shearer F.M., Johnson K. (2019). The current and future global distribution and population at risk of dengue. Nat. Microbiol..

[32] Camargo C., Alfonso-Parra C., Diaz S., Rincon D.F., Ramirez-Sanchez L.F., Agudelo J. (2021). Spatial and temporal population dynmics of male and female *Aedes albopictus* at a local scale in Medellin, Colombia. Parasit. Vec..

[33] Ferreira-de-Lima V.H., Camara D.C.P., Honorio N.A., Lima-Camara T.N. (2020). The Asian tiger mosquito in Brazil: Observations on biology and ecological interactions since first detection in 1986. Acta Trop..

[34] Heinisch M.R.S., Diaz-Quijano F.A., Chiaravalloti-Neto F., Pancetti F.G.M., Coelho R.R., Dos Santos Andrade P. (2019). Seasonal and spatial distribution of *Aedes aegypti* and *Aedes albopictus* in a municipal urban park in Sao Paulo, SP, Brazil. Acta Trop..

[35] Reiskind M.H., Lounibos L.P. (2013). Spatial and temporal patterns of abundance of *Aedes aegypti* L. (*Stegomyia aegypti*) and *Aedes albopictus* (Skuse) [*Stegomyia albopictus* (Skuse)] in southern Florida. Med. Vet. Entomol..

[36] Chadee D.D. (1988). Effects of ‘closed’ houses on the *Aedes aegypti* eradication programme in Trinidad. Med. Vet. Entomol..

[37] Stewart A.T.M., Winter N., Igiede J., Hapairai L.K., James L.D., Feng R.S., Mohammed A., Severson D.W., Duman-Scheel M. (2020). Community acceptance of yeast interfering RNA larvicide technology for control of *Aedes* mosquitoes in Trinidad. PLoS ONE.

[38] Winter N., Stewart A.T.M., Igiede J., Wiltshire R.M., Hapairai L.K., James L.D., Mohammed A., Severson D.W., Duman-Scheel M. (2021). Assessment of Trinidad community stakeholder perspectives on the use of yeast interfering RNA-baited ovitraps for biorational control of *Aedes* mosquitoes. PLoS ONE.

[39] Barrera R., Amador M., Munoz J., Acevedo V. (2018). Integrated vector control of *Aedes aegypti* mosquitoes around target houses. Parasit. Vec..

